# Formic Acid Pursues Efficient Hydrodeoxygenation of Naphthols and Phenolic Derivatives to Arenes

**DOI:** 10.1002/cssc.202502031

**Published:** 2025-12-08

**Authors:** Benedetta Di Erasmo, Edoardo Bazzica, Giulia Brufani, Luigi Vaccaro, Chao‐Jun Li

**Affiliations:** ^1^ Laboratory of Green S.O.C.–Dipartimento di Chimica, Biologia e Biotecnologie Università degli Studi di Perugia Perugia Italy; ^2^ Department of Chemistry, and FRQNT Centre for Green Chemistry and Catalysis McGill University Montreal Canada; ^3^ Dipartimento di Ingegneria Civile dell’Energia dell’Ambiente e dei Materiali Università degli Studi Mediterranea di Reggio Calabria Reggio Calabria Italy

**Keywords:** arenes, formic acid, heterogeneous catalysts, hydrodeoxygenation, LOHCs, naphthols, phenols

## Abstract

The use of high‐pressure hydrogen poses significant challenges, including safety risks, storage problems, and elevated costs. Consequently, developing reductive chemical processes utilizing low‐pressure hydrogen is highly appealing for industrial‐scale applications. This emphasizes the critical need for sustainable alternatives that offer safer and more accessible reaction conditions. Liquid organic hydrogen carriers (LOHCs) are appealing materials due to their capability to generate hydrogen in situ, which can be directly utilized to produce target biofuel precursors, fuels, or fuel additives. Hydrodeoxygenation (HDO) is an efficient approach for transforming lignin and its phenolic derivatives into valuable aromatic chemicals and fuels. Herein, we present an alternative HDO of naphthols and phenols using a commercial heterogeneous catalyst, Pd/C, employing formic acid as LOHC. This paper presents a consistent substrate scope for the HDO of different naphthols and phenols, including pharmaceutically relevant molecules such as amylmetacresol and menthol.

## Graphical abstract

An alternative hydrodeoxygenation of naphthols and phenols using a commercial heterogeneous catalyst, Pd/C, employing formic acid as LOHC is reported. We outline a substrate scope for the HDO of different naphthols and phenols, including pharmaceutically relevant molecules such as amylmetacresol and thymol.

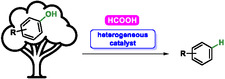

## 1 Introduction

Lignocellulosic biomass, composed primarily of lignin, cellulose, and hemicellulose, represents the most abundant source of non‐food‐based renewable carbon [1, 2, 3]. Its efficient upgrading to fuels and chemicals holds significant potential for reducing the global carbon footprint [4, 5, 6, 7]. The derived platform compounds from lignocellulosic biomass feature desirable chemical linkages, such as furanic and phenyl rings, which provide excellent starting points for various transformations. However, one of the major challenges in biomass valorization lies in its high oxygen content [8, 9, 10, 11]. This is problematic for producing fuels and fuel additives, which require reduced oxygen levels to achieve desirable characteristics. Lignin, often burned in biorefineries for energy and heat, has recently garnered attention for its potential to be upgraded into more valuable products [6, 12, 13, 14, 15, 16, 17, 18, 19, 20]. Numerous efforts have thus focused on developing processes that increase the hydrogen‐to‐carbon (H/C) ratio of the products while reducing their oxygen content [21, 22, 23, 24, 25, 26, 27], and depolymerizing it to phenolic monomers [28, 29].

Phenol derivatives are ubiquitous reagents in chemistry which can provide a wide range of products through different functionalizations [21, 30, 31, 32]. A key reaction that they can undergo is the hydrodeoxygenation (HDO) to arenes [4, 33, 34, 35, 36, 37]. Arenes are key compounds that serve as building blocks for numerous fine chemicals, including fuels, textiles, pharmaceuticals, and paper products. Conventional arenes synthesis methods from phenols require high temperatures and molecular hydrogen, making them challenging in terms of safety due to the high fugacity of H_2_ [38, 39, 40, 41, 42]. To overcome this problem, we recently worked on phenols’ HDO employing hydrazine as a dual reagent, as a reductant and to form the hydrazone intermediate, without employing molecular hydrogen [43]. Alternative strategies include introducing auxiliary functional groups on the phenolic oxygen to create better leaving groups [44] or using catalytic transfer hydrogenation (CTH) with liquid organic hydrogen carriers (LOHCs) instead of molecular H_2_ [25, 45, 46, 47].

LOHCs are small organic molecules, such as formic acid (HCOOH), acetic acid, oxalic acid, methanol, and 2‐propanol, that can release hydrogen to reactants on the catalytic sites [25, 45]. CTH with LOHCs mostly employs metal catalysts, and it is crucial for them to have a high absorbing capacity of hydrogen and acceptors [48]. Using *i*‐PrOH as the hydrogen donor in HDO of *p‐*cresol, in 2017, Wang and Xia group showed the efficient conversion to toluene at 230°C with a Ru/Nb_2_O_5_‐SiO_2_ catalyst [49]. In the same year, Z. Wang et al*.* studied the HDO of phenol over metal/MCM41 (M = Ru, Pd, Pt) in the presence of different hydrogen donors, finding that the best solution is using Ru/MCM41 with MeOH to obtain benzene (73.3% phenol conversion with 26.3% selectivity to the desired product) [50]. In another work, the same group further studied the hydrogenation of phenol employing Ni/Al_2_O_3_ as catalyst and MeOH as the LOHC. Their analysis confirmed that cyclohexanone and cyclohexanol are the predominant liquid products, providing the catalytic efficacy of the Ni‐based catalyst [51]. A pivotal work worth to mention is the CTH promoted by Ni‐Raney and zeolite H‐BEA that converts bio‐oil and lignin directly into arenes in presence of *i*‐PrOH by Rinaldi's group [52].

**SCHEME 1:**
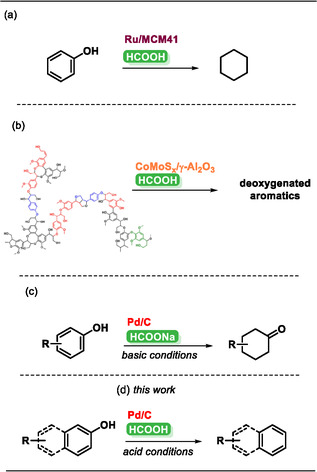
Possible uses of HCOOH for the HDO of lignin and/or phenolic derivatives. (a) Wang et al., Chemical Engineering Journal 2017, 320, 55–62. (b) Kim et al., Chemical Engineering Journal 2018, 348, 799–810. (c) Vaccaro et al., ChemCatChem 2018, 10, 1277–1281. (d) This work.

In recent years, HCOOH has emerged as a viable alternative to molecular hydrogen as an energy carrier in various hydrogenation reactions, owing to its high hydrogen content, ease of storage, and safer handling under ambient conditions [8, 53, 54, 55, 56]. It can be derived from biomass through catalytic processes such as hydrothermal carbonization or bio‐refinery techniques, where lignocellulosic materials, carbohydrates, or organic waste undergo selective oxidation or fermentation [57, 58, 59, 60]. Formic acid has been formerly employed in different hydrogenations of phenols and lignin‐derived compounds (Scheme 1) [61, 62, 63]. Wang et al*.*, as mentioned before, performed a reaction employing Ru/C: wherein HCOOH is more suitable to form cyclohexane (73.9% of phenol conversion with 43.7% selectivity to the desired product) (Scheme 1a) [50]. HCOOH was used by the group of Kim to perform the hydrodeoxygenation (HDO) of a lignin‐derived bio‐oil (from a first depolymerization of sulfonated lignin) employing a CoMo‐based catalyst (Scheme 1b) [64]. They assess 6.8 wt% of deoxygenated compound yield with respect to the initial lignin value and a degree of deoxygenation of > 90%. In 2018, our group proposed a selective hydrogenation of phenol to cyclohexanone through a flow protocol, assessing an important trademark for polymer industry, since cyclohexanone is the precursor of Nylon‐6 and Nylon 6,6 (Scheme 1c) [65]. This paved the way for other works in which various potential catalysts have been used to obtain cyclohexanone [66, 67, 68, 69, 70, 71].

However, there are still no relevant examples where HCOOH has been used to obtain arene derivatives through HDO. Aiming at this goal and inspired by these previous examples, herein, we propose a Pd/C‐catalyzed HDO of 2‐naphthols and phenol derivatives using HCOOH as a hydrogen donor. Given the inherent difficulty of selectively cleaving the C—O bond in phenols without affecting the aromatic ring, this transformation remains mechanistically challenging yet highly valuable. The efficient conversion of phenols to arenes is crucial for producing renewable aromatic hydrocarbons and fine chemicals. The catalytic carbonaceous support exhibits strong hydrogen affinity, facilitating the CTH mechanism. We achieved strong results in the HDO of different substrates, including amylmetacresol and thymol, two compounds of significant pharmaceutical relevance.

## 2 Results and Discussion

We initiated our investigation by screening various reducing agents (see Table S1). Formic acid delivered the best results in the model reaction involving 2‐naphthol with Pd/C as the catalyst and 1,4‐dioxane as the solvent. The plausible mechanism is the one proposed via a metal hydride route: formic acid can undergo decomposition and reforming reactions to produce molecular hydrogen, which remains attached to the catalytic support [50]. In addition to serving as a hydrogen source, formic acid provides the optimal level of acidity required for the final step (differently from the other formate salts in Table S1), in which TFA promotes the dehydration of a water molecule. Also, in the absence of Pd/C, no reaction occurred (Table 1, **entry 1**). We initiate the optimization of the process by screening different amounts of TFA: less than 1 equiv results in lower conversion or no conversion in the absence of TFA (**entries 2–5**), while an over‐stoichiometric amount leads to a drastic reduction, proceeding to tetralin formation (**entries 6–7**). Changing the organic acid (**entries 8–9**) revealed the crucial role played by TFA, ensuring a proper acidic pH value. The role of TFA corresponds to general acid catalysis and its organic nature is essential, so it is completely soluble in the reaction medium. Moreover, the acid strength plays a crucial role in the final dehydration step of the mechanism (see later in the text), which requires sufficiently acidic conditions to proceed efficiently.

**TABLE 1:** Screening hydrodeoxygenation reaction conditions using HCOOH.^a^

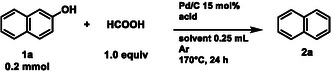			
Entry	Acid	Solvent	**2a,** % b
1^c^	TFA 1 equiv	Dioxane	n.d.
2	—	Dioxane	n.d.
3	TFA 0.5 equiv	Dioxane	Traces
4	TFA 0.8 equiv	Dioxane	56
5	TFA 1 equiv	Dioxane	71
6	TFA 1.5 equiv	Dioxane	42^d^
7	TFA 2 equiv	Dioxane	traces^d^
8	*p*‐TSA 1 equiv	Dioxane	n.d.
9	AcOH 1 equiv	Dioxane	32
10^e^	TFA 1 equiv	Dioxane	35
11	TFA 1 equiv	Toluene	39
12	TFA 1 equiv	CPME	n.d.
13	TFA 1 equiv	Water	n.d.

a Reaction conditions: **1a** (0.2 mmol), Pd/C (5 wt%) 15 mol%, acid, reaction medium 0.25 mL, 24 h, 170°C, Ar, 10 mL pressure tube.

b NMR yields are reported using dibromomethane as the external standard. The remaining percentage is unreacted 2‐naphthol.

c Pd/C 0 mol%, blank test

d Tetralin byproduct is formed.

e 140°C.

Additionally, a decrease in temperature is detrimental to the reaction, resulting in yields of only 35% (**entry 10**). Changing the solvent (**entries 11–13**) did not give any improvement in the reaction, especially with water, which completely inhibits the reaction. The best result, presented in **entry 5**, achieved a 71% NMR yield and a 64% isolated yield, with no detectable byproducts and streamlined product isolation.

To evaluate the generality of the transformation, we next explored the scope of 2‐naphthol derivatives as substrates (Table 2). Substituted 2‐naphthols were readily converted into the corresponding polycyclic aromatic hydrocarbons in consistently high yields. 3‐Methyl‐2‐naphthol (**1b**) underwent smooth conversion to 2‐methylnaphthalene (**2b**) in 76% yield. 6‐Methyl‐2‐naphthol (**1c**) satisfactorily yielded product **2b** in 63%. 8‐Methyl‐2‐naphthol (**3e**) afforded 1‐methylnaphthalene (**4a**) in 68% yield, similarly to 1‐methyl‐2‐naphthol (**1d**) whose 60% has been converted. Notably, benzylic and aryl substituents were also well tolerated under the reaction conditions. Thus, 4‐benzylphenol (**3f**) furnished diphenylmethane (**4f**) in 61% yield, and 4‐phenylphenol (**3g**) afforded the corresponding biphenyl derivative (**4g**) in 65% yield. 4‐Propylphenol (**3h**) was also tested to evaluate the potential applicability of bio renewable phenolic compounds, affording a 33% NMR yield. We further assessed the synthetic utility of the protocol by applying it to pharmaceutically relevant compounds. Both thymol (**1i**) [72], and amylmetacresol (**1l**), widely used as bioactive scaffolds, underwent the transformation to deliver the corresponding products in synthetically useful yields. The main limitation of this protocol arises with halogenated compounds, which undergo dehalogenation without subsequent hydrodeoxygenation to the corresponding arene (Scheme S1). 3‐ and 4‐Methoxyphenols afford the corresponding arenes in 15% yield which can let us conclude that the additional oxygen substituent increases the electron density of the aromatic ring hindering its reduction and the initiation of the mechanism.

**TABLE 2:** CTH‐promoted hydrodeoxygenation of different 2‐naphthols and phenols with HCOOH.^a^

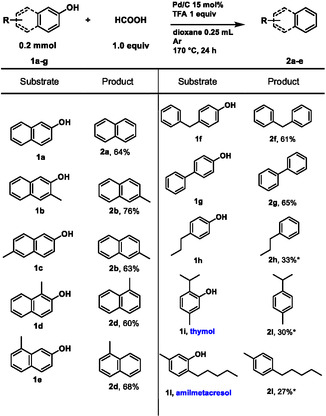

a Reaction conditions: **3a‐g** (0.2 mmol), HCOOH 1.0 equiv, Pd/C (5 wt%) 15 mol%, TFA 1.0 equiv, dioxane 0.25 mL, 24 h, 170°C, Ar, 10 mL pressure tube. Isolated yields are shown; * = NMR yield.

**FIGURE 1:**
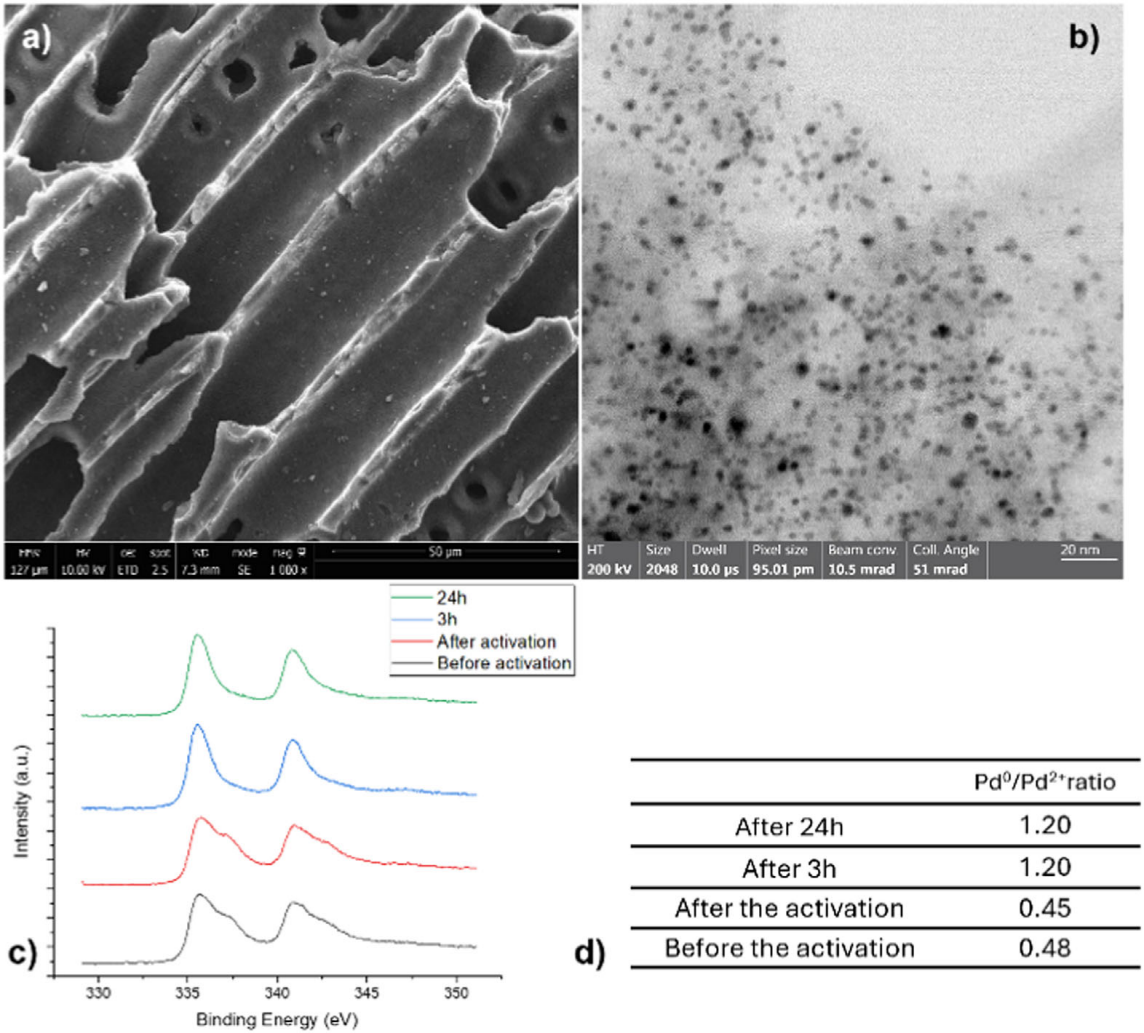
(a) SEM and (b) TEM images after 24 h reaction; (c) Pd 3d XPS analyses at various stages of the reaction; (d) Pd^0^/Pd^2+^ratio.

To check whether the morphology and activity of the catalyst remained stable during the reaction, we performed analysis on the catalysts at different stages of the reaction: before and after the activation, after 3 h and after 24 h (Figure 1 and Supporting Information). SEM and TEM analyses (Figure 1a,b) indicate that the size and shape of Pd nanoparticles remain unchanged in the middle of the reaction (after 3 h) and at the end of the reaction (after 24 h). XPS analysis reveals that activation under vacuum at 140°C is necessary only to remove the water content in the catalyst and to facilitate the dispersion of the nanoparticles; indeed, there is no significant change in the Pd oxidation state before and after activation. We observe a significant increase in the Pd^0^/Pd^2+^ ratio as the reaction progresses: at 3 h (blue line, Figure 1c,d), Pd is completely activated (all Pd^0^) and remains unchanged after 24 h. This change in the oxidation state hinders the recyclability of the catalyst (15% yield of naphthalene upon reuse).

**SCHEME 2:**
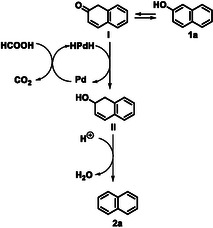
Proposed mechanism.

To gain a deeper understanding of the reaction mechanism, we conducted kinetic studies (Table S2). It became evident that during the first few hours, no intermediates are present in the reaction mixture other than the product itself and the reagent. 2‐naphthol cannot form naphthyl formate with formic acid (pK_a_ TFA = 0.23 ≪ pK_a_ 2‐naphthol = 9.5). Such a mechanism would be feasible only under basic conditions. Indeed, when HCOONa was used (entry 3, Scheme S1), only *α*‐tetralone was obtained, consistent with our previous findings [65, 70]. We suppose then that the plausible mechanism of our reaction is *via* a metal hydride route (Scheme 2) [25]. Firstly, Pd‐mediated decomposition of formic acid to CO_2_ and H_2_ occurs. Molecular hydrogen remains on the surface of Pd/C ready to catalyze the acid‐mediated hydrodeoxygenation [50]. In this mechanism, there is not a complete reduction to tetralone [73]. For this reason, we hypothesize that the carbonyl group of the tautomeric form of the naphthols (**I**) is hydrogenated to yield intermediate (**II**), which subsequently undergoes dehydration, promoted by acidic species (TFA), to give naphthalene (**2a**).

## 3 Conclusion

In summary, we report the first application of formic acid in conjunction with Pd/C for the selective hydrodeoxygenation of naphthols and phenols to arenes *via* catalytic transfer hydrogenation. This innovative, hydrogen‐free approach employs HCOOH as the hydrogen donor. We propose a plausible metal–hydride mechanism: formic acid undergoes catalytic decomposition to release CO_2_ and H_2_, with the latter remaining adsorbed on the Pd/C surface and facilitating substrate reduction. The acidic species promoted the dehydration. The substrate scope demonstrates the effective conversion of various naphthols and phenols into arenes, with isolated yields reaching up to 76%. Characterization by TEM and SEM indicates that the nanoscale morphology of the Pd nanoparticles is preserved. XPS analysis reveals an increased Pd^0^/Pd^2+^ ratio, confirming the formation of Pd^0^ at the conclusion of the catalytic cycle.

## 4 Experimental Section

### 4.1 General Procedure for the Hydrodeoxygenation of Phenols With Formic Aacid (Run at McGill University)

In an oven‐dried 10 mL Schlenk pressure tube, equipped with a magnetic stir‐bar, Pd/C (5 wt%, 15 mol%, 0.03 mmol, 61.4 mg) is added. Then, the tube is sealed with a rubber septum, linked to a high‐vacuum pump and heated at 140°C for 1 h to activate the catalyst. Afterwards, phenolic compound (0.2 mmol) is added under argon and three cycles of evacuation/backfill with argon are performed. Subsequently, dioxane (0.25 mL), HCOOH (1.0 equiv, 0.2 mmol, 7.5 μL), and TFA (1 equiv., 0.2 mmol, 15.4 μL) are added to the mixture under Ar. The vessel is then heated at 170°C for 24 h under stirring. At the end of the reaction, the mixture is passed through a pad of silica gel to remove the heterogeneous catalyst with EtOAc. The residue is purified by chromatographic column or by preparative thin layer chromatography (TLC) using a variable ratio eluent mixture of ETP and EtOAc.

### 4.2 General Procedure for the Hydrodeoxygenation of Phenols With Formic Acid (Run at the University of Perugia)

In a round‐bottom flask equipped with a magnetic stirrer, Pd/C (5 wt%, 400 mg) was dispersed in 10 mL of ethanol absolute and kept under stirring at 65°C. Then, 7 eq. of HCOONa (12.8 mg) were dissolved in 5 mL of ethanol absolute and added dropwise into the Pd/C dispersion and the mixture was stirred at 65°C for 2 h. Once finished, the Pd/C was filtered, washed with water, and dried under vacuum at 130°C overnight [74]. In an 8 mL vial equipped with a magnetic stirrer, the phenolic compound (0.2 mmol) and Pd/C (15 mol%) were added then 3 cycle of vacuum‐argon were performed in order to make the argon atmosphere. Under a flow or Argon, anhydrous 1,4‐dioxane (0.25 mL), HCOOH (1 eq., 7.5 μL), and TFA (1 eq., 15.4 μL) were added and the reaction was performed at 170° for 24 h. Once the reaction is finished, the reaction mixture is filtered on a silica pad to remove the catalyst. The residue is purified by chromatographic column or by preparative thin layer chromatography (TLC) using a variable ratio eluent mixture of ETP and EtOAc.

## Supporting Information

Additional supporting information can be found online in the Supporting Information section. **Supporting** **Scheme** **S1:** Unsuccessful substrates. **Supporting** **Fig.** **S1:** TEM (a) and SEM (b) analyses of the commercial Pd/C catalyst before the activation. **Supporting** **Fig.** **S2:** TEM (a) and SEM (b) analyses of the activated Pd/C. No change in morfology and size is observed after the activation, possibly indicating that the activation only affects the water content of the catalyst. **Supporting** **Fig.** **S3:** TEM (left:bright field,right: HAADF) (a) and SEM (b) analyses of Pd/C after 3h reaction time. During the reaction, no particular change in morfology and size is observed. **Supporting** **Fig.** **S4:** TEM (a) and SEM (b) analyses of Pd/C after 24h reaction time. After the reaction, no change in morfology and size is observed. The catalyst mantains its properties. **Supporting** **Table S1:** Preliminary optimization of the reaction conditions. **Supporting** **Table S2:** Kinetic studies.

## Funding

The work was supported by Canada Research Chairs and European Commission (Grant ECS00000041); Canada Foundation for Innovations; Natural Sciences and Engineering Research Council of Canada; Fonds de recherche du Québec – Nature et technologies (FRQNT).

## Conflicts of Interest

The authors declare no conflicts of interest.

## Supporting information

Supplementary Material

## Data Availability

The data that support the findings of this study are available from the corresponding author upon reasonable request.
