# Supplementary material for: Formic Acid Pursues Efficient Hydrodeoxygenation of Naphthols and Phenolic Derivatives to Arenes
Source: ChemSusChem. 2025 Dec 8;19(1):e202502031. doi: 10.1002/cssc.202502031 (PMC12767554; doi:10.1002/cssc.202502031)

SUPPORTING INFORMATION FILE

Formic Acid pursues efficient Hydrodeoxygenation of Naphthols and Phenolic derivatives to arenes

Benedetta Di Erasmo,[a,b] Edoardo Bazzica,[a] Giulia Brufani,[a] Luigi Vaccaro,[a] * Chao-Jun Li[b] *

[a] *Laboratory of Green S.O.C. – Dipartimento di Chimica, Biologia e Biotecnologie, Università degli Studi di Perugia, Via Elce di Sotto 8, 06123, Perugia, Italy*.

[b] *Department of Chemistry, and FRQNT Centre for Green Chemistry and Catalysis – McGill University, 801 Sherbrooke Street West, Montreal, QC H3A0B8, Canada*

e-mail: luigi.vaccaro@unipg.it

e-mail: cj.li@mgill.ca

Table of contents:

1. General Remarks ……………………………………………………...………………..…S2
2. Experimental Procedures………………………………………………….……….............S3
   2.1 General procedure for the hydrodeoxygenation of phenols with formic acid (run at McGill University……………...………………………………………….……….................S3
   2.2. General procedure for the hydrodeoxygenation of phenols with formic acid (run at the University of Perugia) ……………...……………………………………….……….........S4
3. Preliminary optimization ………………………….......……......…......……..................S5
4. Kinetic studies……………………......……………………........…..………..……...…................S5
5. Scope of the reaction…..……......……………………........…..………..……...…................S6
6. TEM and SEM analyses………..……..........….........….........…..........…......................S6
7. Spectral data of isolated compounds……..........…........................…......……….........S9
8. ^{1}H-NMR and ^{13}C-NMR spectra of isolated compounds……...........…........………........S16

1. General Remarks

All reactions were carried out under an atmosphere of argon, unless otherwise stated. Solvents and reagents were purchased from Sigma-Aldrich and Ambeed chemical companies and were used without further purification unless otherwise specified. 1,4-Dioxane was purified by the Pure Solvent MD-7 purification system (Innovative Technology). Product purifications were performed either with column chromatography on a Biotage Isolera One automated chromatography system on silica gel or with preparative analytical thin-layer chromatography (TLC) using E. Merck silica gel 60 F254 pre-coated plates (0.25 mm). Nuclear magnetic resonance (^{1}H and ^{13}C) spectra were recorded on a Bruker AV500 equipped with a 60-position Sample Xpress sample changer (^{1}H, 500 MHz, 400 MHz; ^{13}C, 125 MHz, 100 MHz). Chemical shifts are expressed in parts per million (ppm) units downfield from TMS, with the solvent residue peak as the chemical shift standard ($CDCl_3$: δ 7.26 ppm in ^{1}H NMR,

δ 77.16 ppm in ^{13}C NMR; DMSO-d6 δ 2.50 ppm in ^{1}H NMR, δ 39.52 ppm in ^{13}C NMR; acetone-d6 δ 2.04 ppm in ^{1}H NMR, δ 206.3 ppm in ^{13}C NMR). Data are reported as following: chemical shift, multiplicity (s = singlet, d = doublet, dd = doublet of doublets, t = triplet, td = triplet of doublets, q = quartet, quint = quintet, sext = sextet, sept = septet, m = multiplet, br = broad singlet), coupling constants J (Hz), and integration. All NMR spectra were recorded at room temperature. Initial catalytic tests were analysed with a GC/FID 5975C Agilent series equipped with a capillary column DB-5MS (30 m, 0.32 mm), an FID detector, and helium as a gas carrier. EI-MS was obtained from the Agilent GC-MS system. High-resolution mass spectrometry was conducted by using atmospheric pressure chemical ionization (APCI) or electro-spraying ionization (ESI) performed by McGill University on a Thermo-Scientific Exactive Orbitrap. Protonated/deprotonated molecular ions or sodium adducts were used for empirical formula confirmation. The bright field transmission electron microscopy (TEM) images were obtained on FEI Tecnai G2 F20 S/TEM at accelerating voltage of 200 kV. The high-angle annular dark-field scanning transmission electron microscopy (HAADF-STEM) characterization was carried out on a Hitachi HD2700 Cs-corrected STEM, which was used with a cold field emitter operated at 200 kV and with an electron beam diameter of ~ 0.1 nm. The scanning electron microscopy (SEM) was carried out on a FEI Quanta 450 Environment scanning electron microscopy (FE-ESEM). All reactions are stirred magnetically unless otherwise specified.

2. Experimental procedures

2.1. General procedure for the hydrodeoxygenation of phenols with formic acid (run at McGill University)

In an oven-dried 10 mL Schlenk pressure tube, equipped with a magnetic stir-bar, Pd/C (5 wt%, 15 mol%, 0.03 mmol, 61.4 mg) is added. Then, the tube is sealed with a rubber septum, linked to a high-vacuum pump and heated at 140 °C for 1 h to activate the catalyst. Afterwards, phenolic compound (0.2 mmol) is added under argon and three cycles of evacuation/backfill with argon are performed.

Subsequently, dioxane (0.25 mL), HCOOH (1.0 equiv, 0.2 mmol, 7.5 μL) and TFA (1 equiv., 0.2 mmol, 15.4 μL) are added to the mixture under Ar. The vessel is then heated at 170 °C for 24 h under stirring. At the end of the reaction the mixture is passed through a pad of silica gel to remove the heterogeneous catalyst with EtOAc. The residue is purified by chromatographic column or by preparative thin layer chromatography (TLC) using a variable ratio eluent mixture of ETP and EtOAc.

2.2. General procedure for the hydrodeoxygenation of phenols with formic acid (run at the University of Perugia)

In a round-bottom flask equipped with a magnetic stirrer Pd/C (5 wt%, 400 mg) was dispersed in 10 mL of ethanol absolute and kept under stirring at 65°C. Then 7 eq. of HCOONa (12.8 mg) were dissolved in 5 mL of ethanol absolute and added dropwise into the Pd/C dispersion and the mixture was stirred at 65°C for 2h. Once finished, the Pd/C was filtered, washed with water and dried under vacuum at 130°C overnight (G. Agostini *et al., ACS Catal.* **2014**, *4*, 1, 187–194). In an 8 mL vial equipped with a magnetic stirrer the phenolic compound (0.2 mmol) and Pd/C (15 mol%) were added then 3 cycle of vacuum-argon were performed in order to make the argon atmosphere. Under a flow or Argon, anhydrous 1,4-dioxane (0.25 mL), HCOOH (1 eq., 7.5 μL) and TFA (1 eq., 15.4 μL) were added and the reaction was performed at 170° for 24 h. Once the reaction is finished the reaction mixture is filtered on a silica pad to remove the catalyst. The residue is purified by chromatographic column or by preparative thin layer chromatography (TLC) using a variable ratio eluent mixture of ETP and EtOAc.

3. Preliminary optimization

Table S1. Preliminary optimization of the reaction conditions.[a]

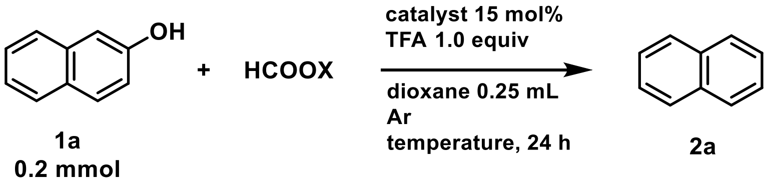

Entry	Catalyst	Formate source	NMR yield of 2a[b] (%)
1	Pd/C (5 wt%)	HCOOH 1.0 equiv	64
2	Pd/C (5 wt%)	HCOOH 1.5 equiv	17[c]
3	Pd/C (5 wt%)	$HCOONH_4$ 3.0 equiv	50
4	Pd/C (5 wt%)	HCOONa 3.0 equiv	0[d]
5	Pd/C (10 wt%)	HCOOH 1.0 equiv	42
6	$Pd(OAc)_2$	HCOOH 2.0 equiv	0

[a] Reaction conditions: **1a** (0.2 mmol), HCOOX**,** Pd 15 mol%, TFA 1.0 equiv, dioxane 0.25 mL, 24 h, 170°C, Ar, 10 mL pressure tube. [b]Obtained using dibromomethane as the I.S. The remaining percentage is unreacted 2-naphthol. [c] 20% is tetralin [d] 50% yield of α-tetralone has been obtained

4. Kinetic studies

Table S2. Kinetic studies.

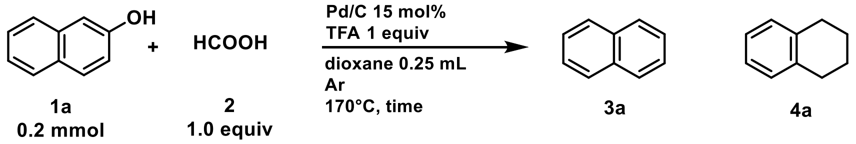

Entry	Time	1a	3a	4a
2	15 min	100	0	0
3	1 h	95	5	0
5	3 h	63	37	0
7	10 h	48	52	0
8	14 h	38	60	2
10	24 h	20	71	6
11	41 h	6	52	42

[a] Reaction conditions: **1a** (0.2 mmol), **2 ,** Pd/C 15 mol%, N_2H_4 in THF [1.0 M] 2 equiv., TFA 1 equiv, dioxane 0.25 mL, 24 h, 170°C, Ar, 10 mL pressure tube. NMR yields are reported using dibromomethane as I.S

5. Scope of the reaction

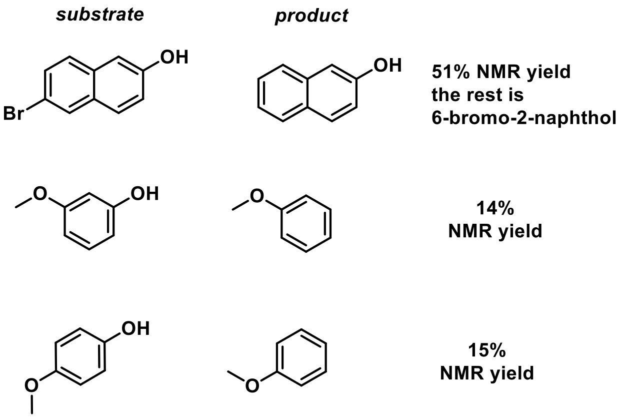

Scheme S1. Unsuccessful substrates.

6. TEM and SEM analyses

Figure S1. TEM (a) and SEM (b) analyses of the commercial Pd/C catalyst before the activation.

a) 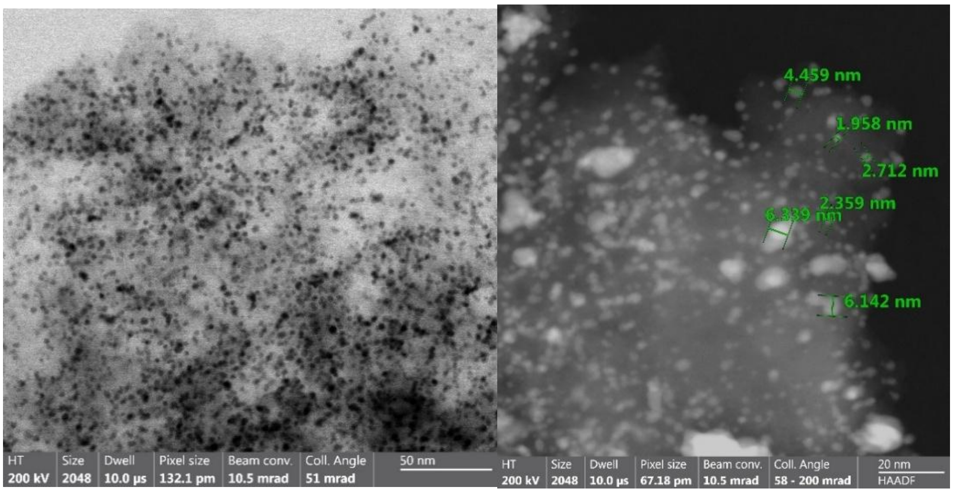

b) 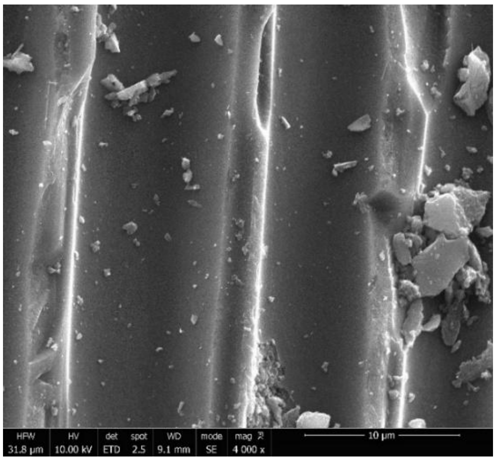

Figure S2. TEM (a) and SEM (b) analyses of the activated Pd/C. No change in morfology and size is observed after the activation, possibly indicating that the activation only affects the water content of the catalyst.

a) 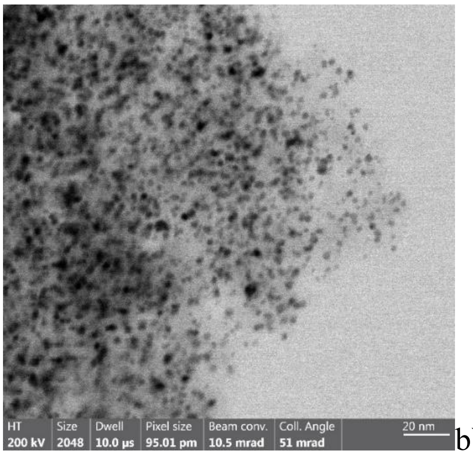

b) 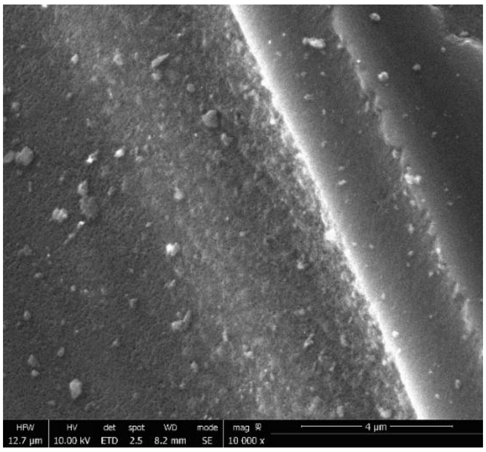

Figure S3. TEM (left:bright field,right: HAADF) (a) and SEM (b) analyses of Pd/C after 3h reaction time. During the reaction, no particular change in morfology and size is observed.

a) 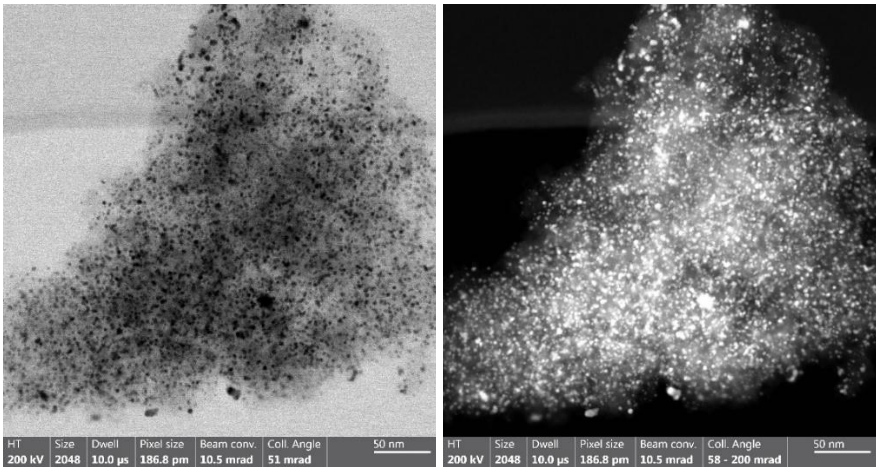

b) 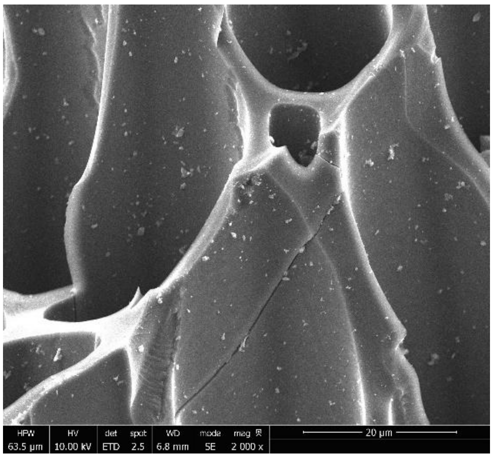

Figure S4. TEM (a) and SEM (b) analyses of Pd/C after 24h reaction time. After the reaction, no change in morfology and size is observed. The catalyst mantains its properties.

a)

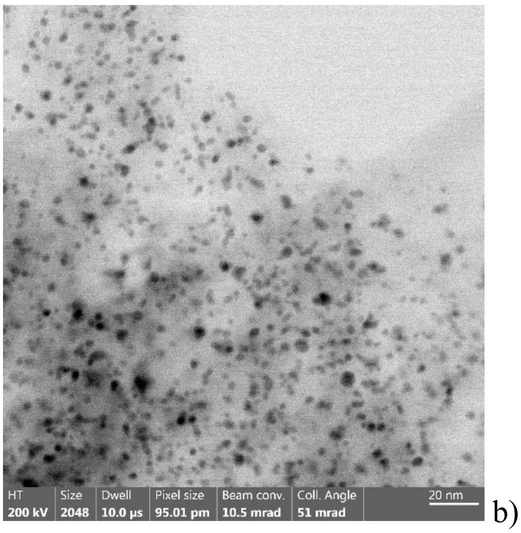

b)

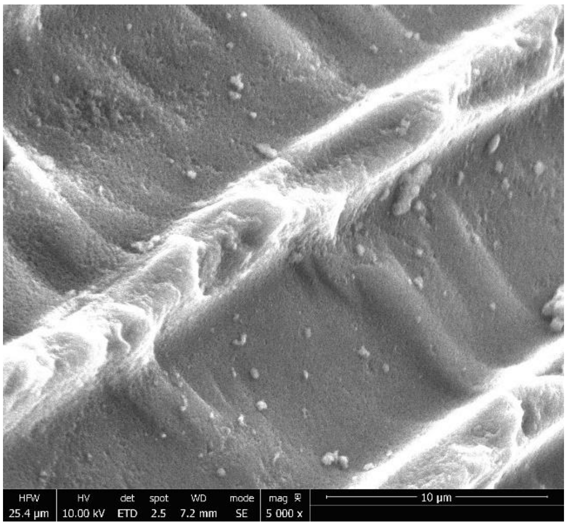

7. Spectral data of isolated compounds

**Chem. Name**	**Naphthalene (2a)**
**Lit. Ref.**	S. Kato, Y. Saga, M. Kojima, H. Fuse, S. Matsunaga, A. Fukatsu, M. Kondo, S. Masaoka, M. Kana, *J. Am. Chem. Soc.* **2017**, *139*, 2204−2207, https://doi.org/10.1021/jacs.7b00253

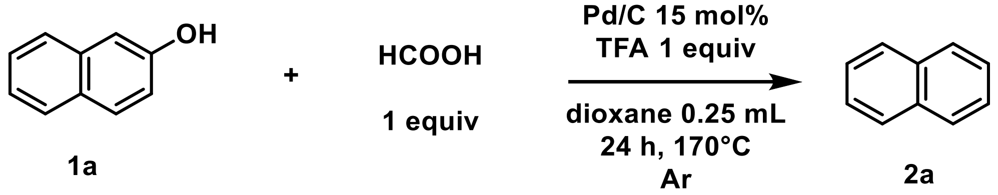

METHOD:

In an oven-dried 10 mL Schlenk pressure tube, equipped with a magnetic stirbar Pd/C (15 wt%, 15 mol%, 0.03 mmol, 61.4 mg) is added. Then, the tube is sealed with a rubber septa and linked to an high-vacuum pump and it is heated at 140 °C for 1 h to activate the catalyst. After 2-naphthol (0.2 mmol, 28.8 mg) is added under Argon and three cycles of evacuation/backfill with Argon are performed. Subsequently, HCOOH (1.0 equiv., 0.2 mmol, 7.3 µL) and TFA (1 equiv., 0.2 mmol, 15.4 µL) are added to the mixture under Ar. The vessel is then heated at 170 °C for 24 h under stirring. At the end of the reaction the mixture is passed through a pad of silica gel to remove the heterogeneous catalyst with EtOAc. The filtrate is then purified with a TLC preparative with pentane as the eluent. The purified product is obtained as a white solid. (isolated yield 64%, 16 mg).

**Mol Formula**	$C_{10}H_8$	**m.p.**	79°C– 80°C	
**^{1}H NMR 500 MHz $CDCl_3$**	**δ value**	**No. H**	**Mult.**	**J value/Hz**
	7.86-7.84	4	*m*	
	7.51-7.48	4	*m*	

^{13}C NMR (126 MHz, $CDCl_3$) δ: 133.5, 127.9, 125.8

GC-EIMS (m/z, %): calculated for 128.0621 (M); found: 128.0625

Chem. Name	2-methyl-naphthalene (2b)
Lit. Ref.	S. Kato, Y. Saga, M. Kojima, H. Fuse, S. Matsunaga, A. Fukatsu, M. Kondo, S. Masaoka, M. Kana, *J. Am. Chem. Soc.* **2017**, *139*, 2204−2207, https://doi.org/10.1021/jacs.7b00253

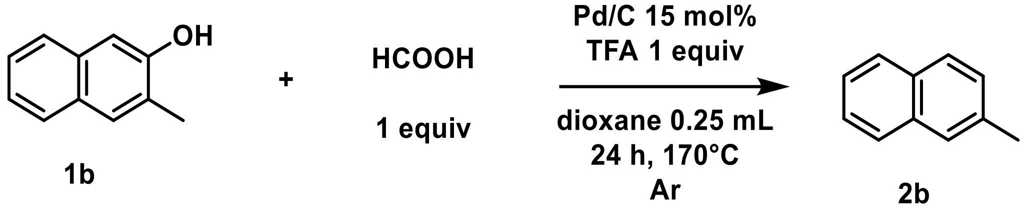

METHOD:

In an oven-dried 10 mL Schlenk pressure tube, equipped with a magnetic stirbar Pd/C (15 wt%, 15 mol%, 0.03 mmol, 61.4 mg) is added. Then, the tube is sealed with a rubber septa and linked to an high-vacuum pump and it is heated at 140 °C for 1 h to activate the catalyst. After 3-methyl-2-naphthol (0.2 mmol, 31.6 mg) is added under Argon and three cycles of evacuation/backfill with Argon are performed. Subsequently, HCOOH (1.0 equiv., 0.2 mmol, 7.3 µL) and TFA (1 equiv., 0.2 mmol, 15.4 µL) are added to the mixture under Ar. The vessel is then heated at 170 °C for 24 h under stirring. At the end of the reaction the mixture is passed through a pad of silica gel to remove the heterogeneous catalyst with EtOAc. The filtrate is then purified with a TLC preparative with hexane as the eluent. The purified product is obtained as a pale yellow liquid. (isolated yield 76%, 22 mg).

**Mol Formula**		$C_{11}H_{10}$	**m.p.**	Pale yellow liquid
**^{1}H NMR 500 MHz $CDCl_3$**	**δ value**	**No. H**	**Mult.**	**J value/Hz**
	7.80	1	*d*	8.0
	7.77-7.74	2	*m*	-
	7.62	1	*s*	-
	7.46-7.40	2	*m*	-
	7.33-7.32	1	*dd*	1.6; 8.4
	2.53	3	*s*	-

^{13}C NMR (126 MHz, $CDCl_3$) δ : 135.5, 133.7, 131.7, 128.1, 127.7, 127.6, 127.2, 126.8, 125.9, 124.9, 21.7

GC-EIMS (m/z, %): calculated for 141.0699 (M+); found: 141.0706

Chem. Name	**2-methyl-naphthalene (2b)**
**Lit. Ref.**	S. Kato, Y. Saga, M. Kojima, H. Fuse, S. Matsunaga, A. Fukatsu, M. Kondo, S. Masaoka, M. Kana, *J. Am. Chem. Soc.* **2017**, *139*, 2204−2207, , https://doi.org/10.1021/jacs.7b00253

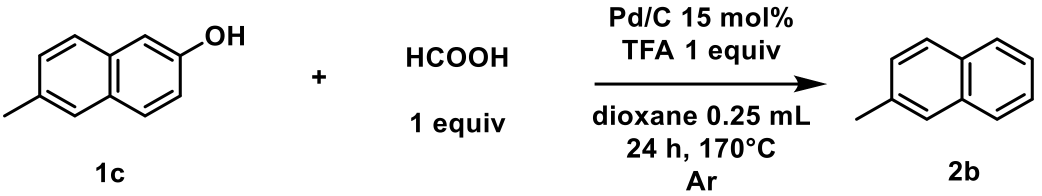

METHOD:

In an oven-dried 10 mL Schlenk pressure tube, equipped with a magnetic stirbar Pd/C (15 wt%, 15 mol%, 0.03 mmol, 61.4 mg) is added. Then, the tube is sealed with a rubber septa and linked to an high-vacuum pump and it is heated at 140 °C for 1 h to activate the catalyst. After 6-methyl-2-naphthol (0.2 mmol, 31.6 mg) is added under Argon and three cycles of evacuation/backfill with Argon are performed. Subsequently, HCOOH (1.0 equiv., 0.2 mmol, 7.3 µL) and TFA (1 equiv., 0.2 mmol, 15.4 µL) are added to the mixture under Ar. The vessel is then heated at 170 °C for 24 h under stirring. At the end of the reaction the mixture is passed through a pad of silica gel to remove the heterogeneous catalyst with EtOAc. The filtrate is then purified with a TLC preparative with hexane as the eluent. The purified product is obtained as a pale yellow liquid. (isolated yield 63%, 18 mg).

**Mol Formula**	$C_{11}H_{10}$	**m.p.**	Pale yellow liquid

	**δ value**	**No. H**	**Mult.**	**J value/Hz**
**^{1}H NMR 500 MHz $CDCl_3$**	7.80	1	*d*	8.0
	7.77-7.73	2	*m*	-
	7.62	1	*s*	-
	7.46-7.39	2	*m*	-
	7.33-7.31	1	*dd*	1.6; 8.4
	2.52	3	*s*	-

^{13}C NMR (126 MHz, $CDCl_3$) δ : 135.4, 133.7, 131.7, 128.1, 127.7, 127.6, 127.2, 126.8, 125.9, 125.0, 21.7

GC-EIMS (m/z, %): calculated for 141.0699 (M+); found: 141.0706

**Chem. Name**	**1-methyl-naphthalene (2d)**
**Lit. Ref.**	S. Kato, Y. Saga, M. Kojima, H. Fuse, S. Matsunaga, A. Fukatsu, M. Kondo, S. Masaoka, M. Kana, *J. Am. Chem. Soc.* **2017**, *139*, 2204−2207; , https://doi.org/10.1021/jacs.7b00253

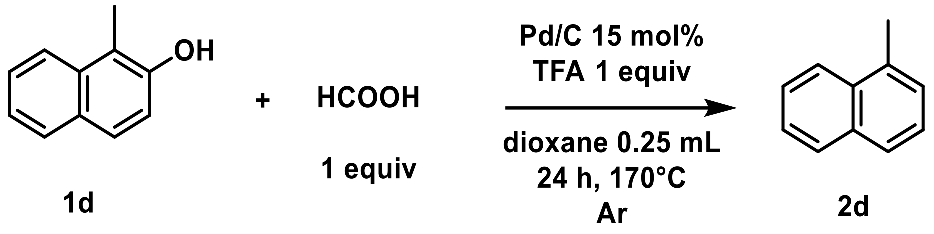

METHOD:

In an oven-dried 10 mL Schlenk pressure tube, equipped with a magnetic stirbar Pd/C (15 wt%, 15 mol%, 0.03 mmol, 61.4 mg) is added. Then, the tube is sealed with a rubber septa and linked to an high-vacuum pump and it is heated at 140 °C for 1 h to activate the catalyst. After 1-methyl-2-naphthol (0.2 mmol, 31.6 mg) is added under Argon and three cycles of evacuation/backfill with Argon are performed. Subsequently, HCOOH (1.0 equiv., 0.2 mmol, 7.3 µL) and TFA (1 equiv., 0.2 mmol, 15.4 µL) are added to the mixture under Ar. The vessel is then heated at 170 °C for 24 h under stirring. At the end of the reaction the mixture is passed through a pad of silica gel to remove the heterogeneous catalyst with EtOAc. The filtrate is then purified with a TLC preparative with pentane as the eluent. The purified product is obtained as a yellow liquid. (isolated yield 60%, 17 mg).

**Mol Formula**		$C_{11}H_{10}$	**m.p.**	Yellow liquid
	**δ value**	**No. H**	**Mult.**	**J value/Hz**
**^{1}H NMR 500 MHz $CDCl_3$**	8.00	1	*d*	8.3
	7.86-7.84	1	*m*	-
	7.71	1	*d*	8.2
	7.54-7.47	2	*m*	
	7.39-7.36	1	*m*	-
	7.33-7.32	1	*m*	-
	2.71	3	*s*	

^{13}C NMR (126 MHz, $CDCl_3$) δ: 134.3, 133.6, 132.6, 128.5, 126.6, 126.4, 125.7, 125.6, 125.5, 124.1, 19.4

GC-EIMS (m/z, %): calculated for 141.0699 (M+); found: 141.0694

Chem. Name	**1-methyl-naphthalene (2d)**
**Lit. Ref.**	S. Kato, Y. Saga, M. Kojima, H. Fuse, S. Matsunaga, A. Fukatsu, M. Kondo, S. Masaoka, M. Kana, *J. Am. Chem. Soc.* **2017**, *139*, 2204−2207, https://doi.org/10.1021/jacs.7b00253

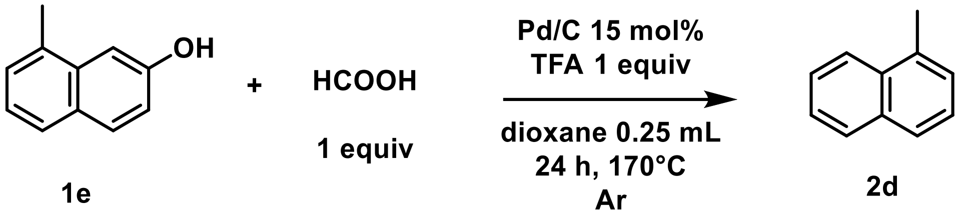

METHOD:

In an oven-dried 10 mL Schlenk pressure tube, equipped with a magnetic stirbar Pd/C (15 wt%, 15 mol%, 0.03 mmol, 61.4 mg) is added. Then, the tube is sealed with a rubber septa and linked to an high-vacuum pump and it is heated at 140 °C for 1 h to activate the catalyst. After 8-methyl-2-naphthol (0.2 mmol, 31.6 mg) is added under Argon and three cycles of evacuation/backfill with Argon are performed. Subsequently, HCOOH (1.0 equiv., 0.2 mmol, 7.3 µL) and TFA (1 equiv., 0.2 mmol, 15.4 µL) are added to the mixture under Ar. The vessel is then heated at 170 °C for 24 h under stirring. At the end of the reaction the mixture is passed through a pad of silica gel to remove the heterogeneous catalyst with EtOAc. The filtrate is then purified with a TLC preparative with pentane as the eluent. The purified product is obtained as a yellow liquid. (isolated yield 68%, 19 mg).

**Mol Formula**		$C_{11}H_{10}$	**m.p.**	Yellow liquid
	**δ value**	**No. H**	**Mult.**	**J value/Hz**
**^{1}H NMR 500 MHz $CDCl_3$**	8.04	1	*d*	8.0
	7.90-7.88	1	*m*	-
	7.75	1	*d*	8.1
	7.58-7.51	2	*m*	
	7.44-7.40	1	*m*	-
	7.37-7.36	1	*m*	-
	2.74	3	*s*	

^{13}C NMR (126 MHz, $CDCl_3$) δ: 134.3, 133.6, 132.7, 128.5, 126.6, 126.4, 125.8, 125.6, 125.6, 124.2, 19.4

GC-EIMS (m/z, %): calculated for 141.0699 (M+); found: 141.0694

Chem. Name	diphenylmethane (2f)
Lit. Ref.	M. Mehta, J. M. Goicoechea; *Angew. Chem. Int. Ed.* **2020**, *59*, 2715 –2719, https://doi.org/10.1002/anie.201915547

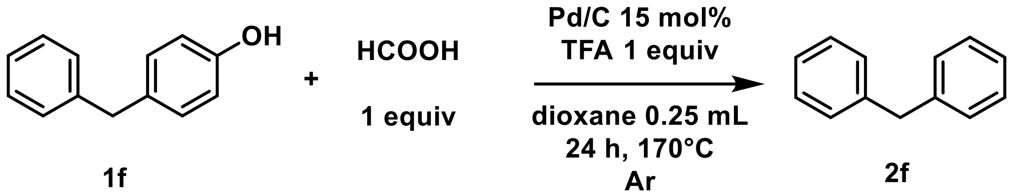

METHOD:

In an oven-dried 10 mL Schlenk pressure tube, equipped with a magnetic stirbar Pd/C (15 wt%, 15 mol%, 0.03 mmol, 61.4 mg) is added. Then, the tube is sealed with a rubber septa and linked to an high-vacuum pump and it is heated at 140 °C for 1 h to activate the catalyst. After 4-benzylphenol (0.2 mmol, 36.8 mg) is added under Argon and three cycles of evacuation/backfill with Argon are performed. Subsequently, HCOOH (1.0 equiv., 0.2 mmol, 7.3 μL) and TFA (1 equiv., 0.2 mmol, 15.4 μL) are added to the mixture under Ar. The vessel is then heated at 170 °C for 24 h under stirring. At the end of the reaction the mixture is passed through a pad of silica gel to remove the heterogeneous catalyst with EtOAc. The filtrate is then purified with a TLC preparative with pentane as the eluent. The purified product is obtained as a white solid. (isolated yield 61%, 21 mg).

**Mol Formula**	$C_{13}H_{12}$	**m.p.**	260°C–264 °C

	**δ value**	**No. H**	**Mult.**	**J value/Hz**
**^{1}H NMR 500 MHz $CDCl_3$**	7.30-7.27	4	*m*	-
	7.21-7.19	6	*m*	-
	3.99	2	*s*	

^{13}C NMR (126 MHz, $CDCl_3$) δ: 141.1, 128.9, 128.5, 126.1, 41.9

GC-EIMS (m/z, %): 169 (M+1, 12), 168 (M, 94), 167 (100), 166 (13), 165 (41), 153 (24), 152 (24), 115 (11), 91 (19), 51 (12)

**Chem. Name**	**1,1’-biphenyl (2g)**
**Lit. Ref.**	G. Ranjani, R. Nagarajan, *Org. Lett.* **2017**, *19*, 15, 3974–3977; https://doi.org/10.1021/acs.orglett.7b01669

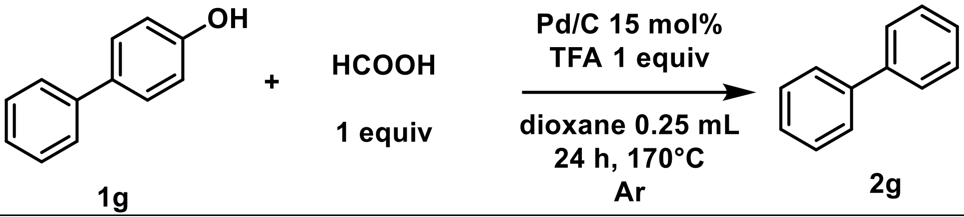

METHOD:

In an oven-dried 10 mL Schlenk pressure tube, equipped with a magnetic stirbar Pd/C (15 wt%, 15 mol%, 0.03 mmol, 61.4 mg) is added. Then, the tube is sealed with a rubber septa and linked to an high-vacuum pump and it is heated at 140 °C for 1 h to activate the catalyst. After 4-phenylphenol (0.2 mmol, 34 mg) is added under Argon and three cycles of evacuation/backfill with Argon are performed. Subsequently, HCOOH (1.0 equiv., 0.2 mmol, 7.3 μL) and TFA (1 equiv., 0.2 mmol, 15.4 μL) are added to the mixture under Ar. The vessel is then heated at 170 °C for 24 h under stirring. At the end of the reaction the mixture is passed through a pad of silica gel to remove the heterogeneous catalyst with EtOAc. The filtrate is then purified with a TLC preparative with pentane as the eluent. The purified product is obtained as a white solid. (isolated yield 65%, 14 mg).

**Mol Formula**	$C_{12}H_{10}$	**m.p.**	67°C–68°C

	**δ value**	**No. H**	**Mult.**	**J value/Hz**
**^{1}H NMR 500 MHz $CDCl_3$**	7.61-7.59	4	*m*	-
	7.46-7.43	4	*m*	-
	7.37-7.34	2	*m*	-

^{13}C NMR (126 MHz, $CDCl_3$) δ: 141.3, 128.8, 127.3, 127.2

GC-EIMS (m/z, %): 155 (M+1,12), 154 (M, 100), 153 (43), 152 (32), 74 (19), 63 (19), 51 (32), 50 (29)

naphthalene

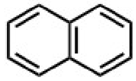

2a

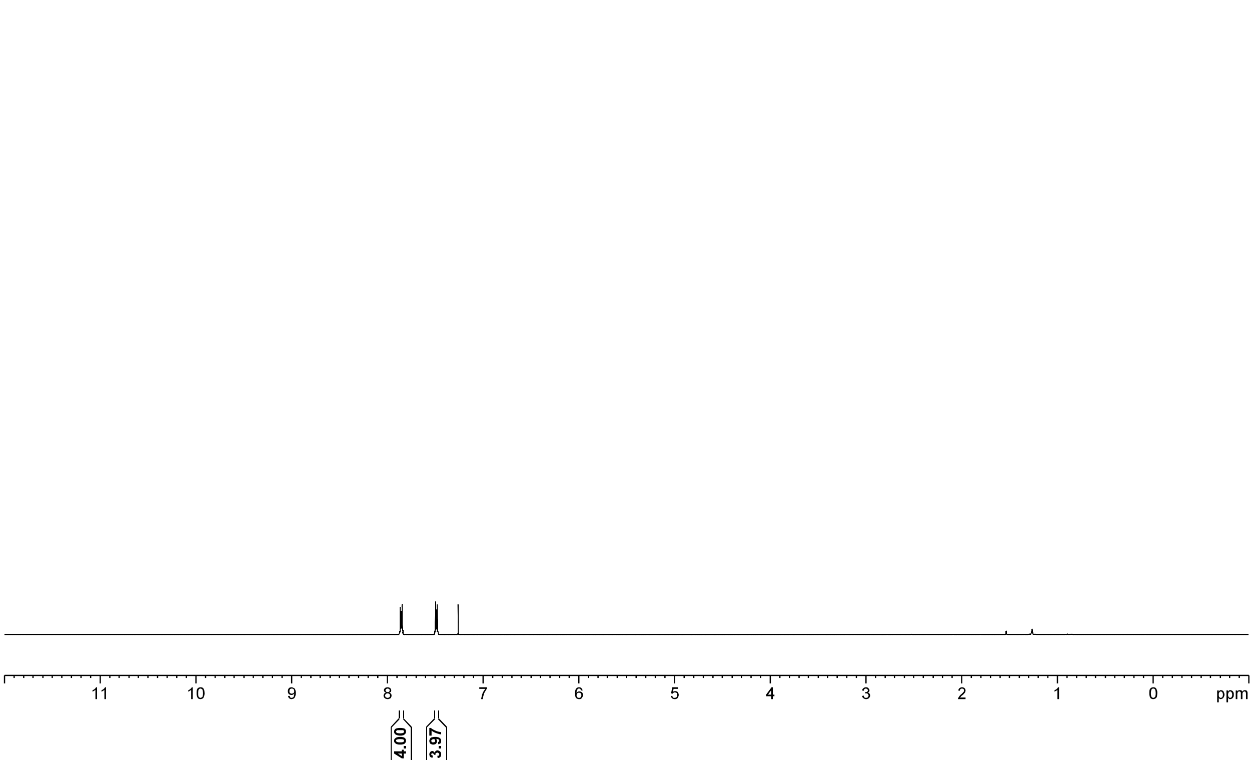

naphthalene

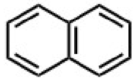

2a

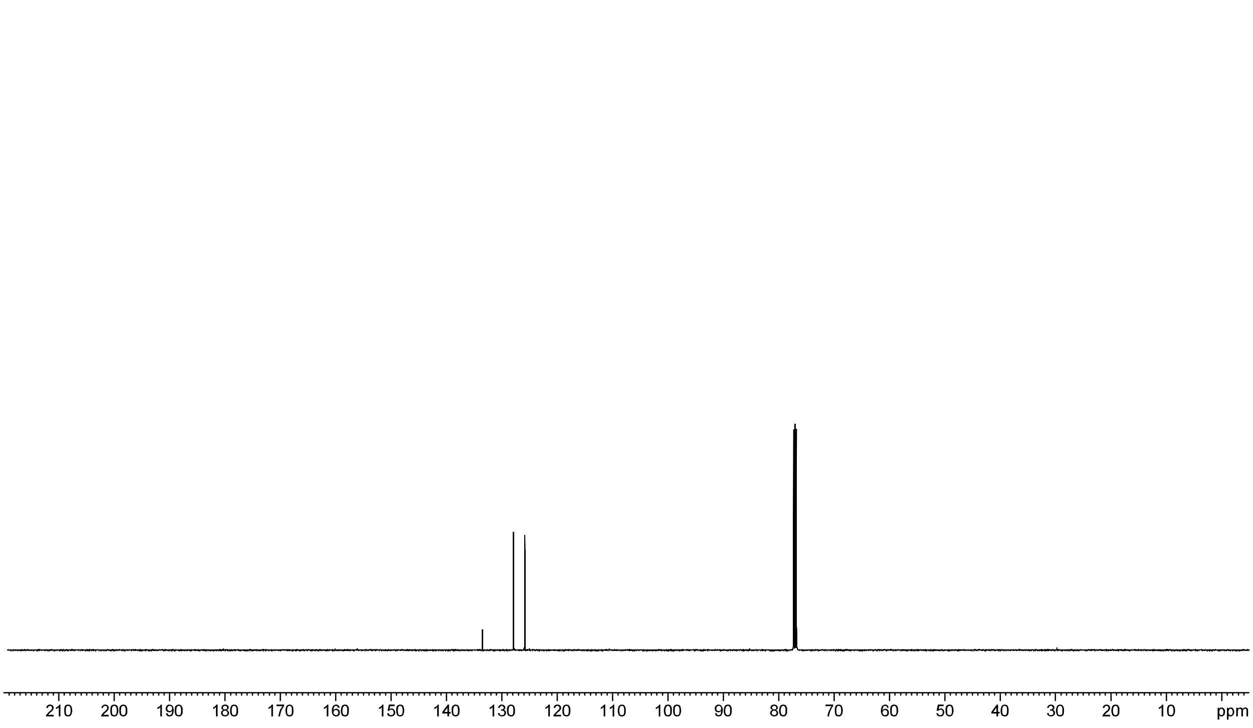

2-methyl-naphthalene

2b

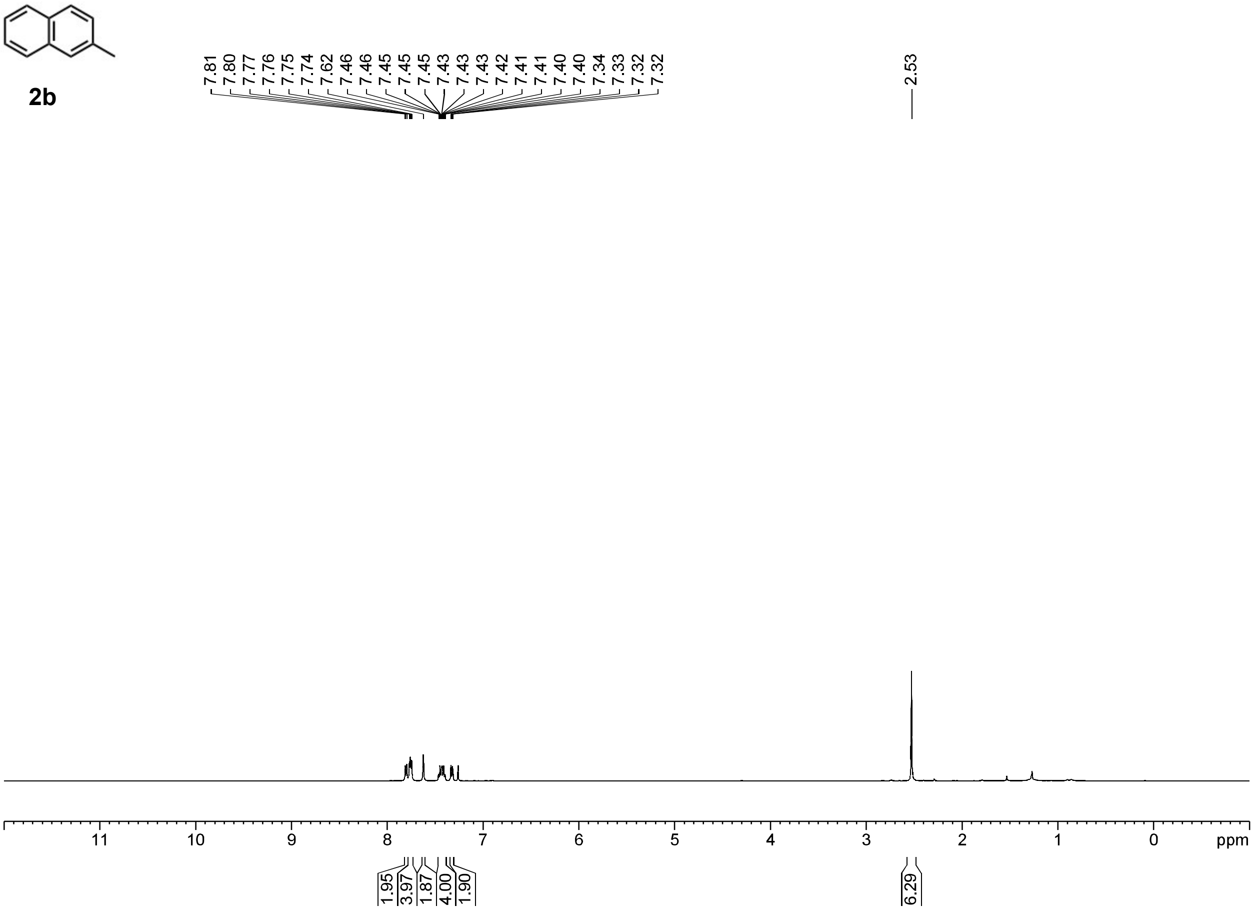

2-methyl-naphthalene

2b

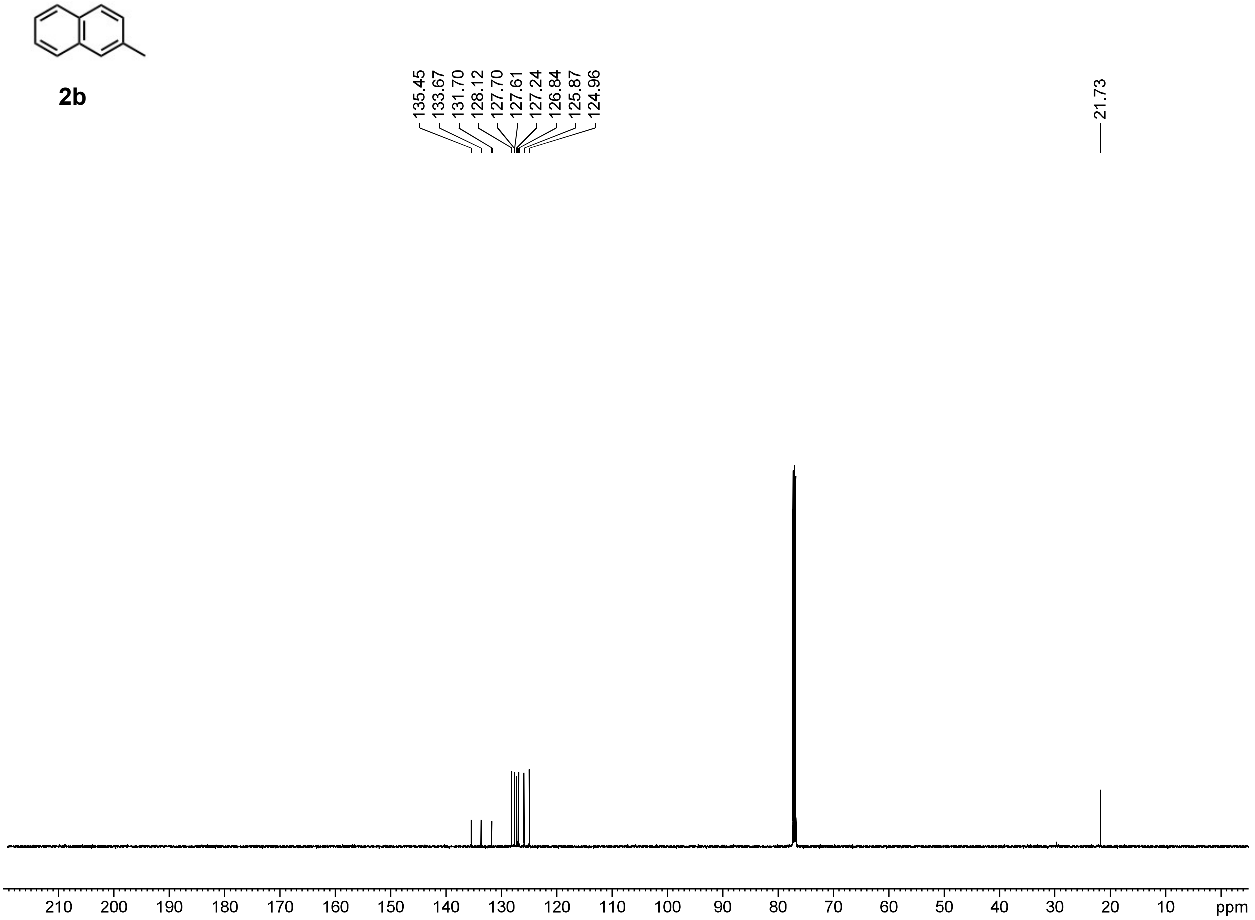

2-methyl-naphthalene

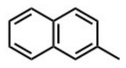

2b

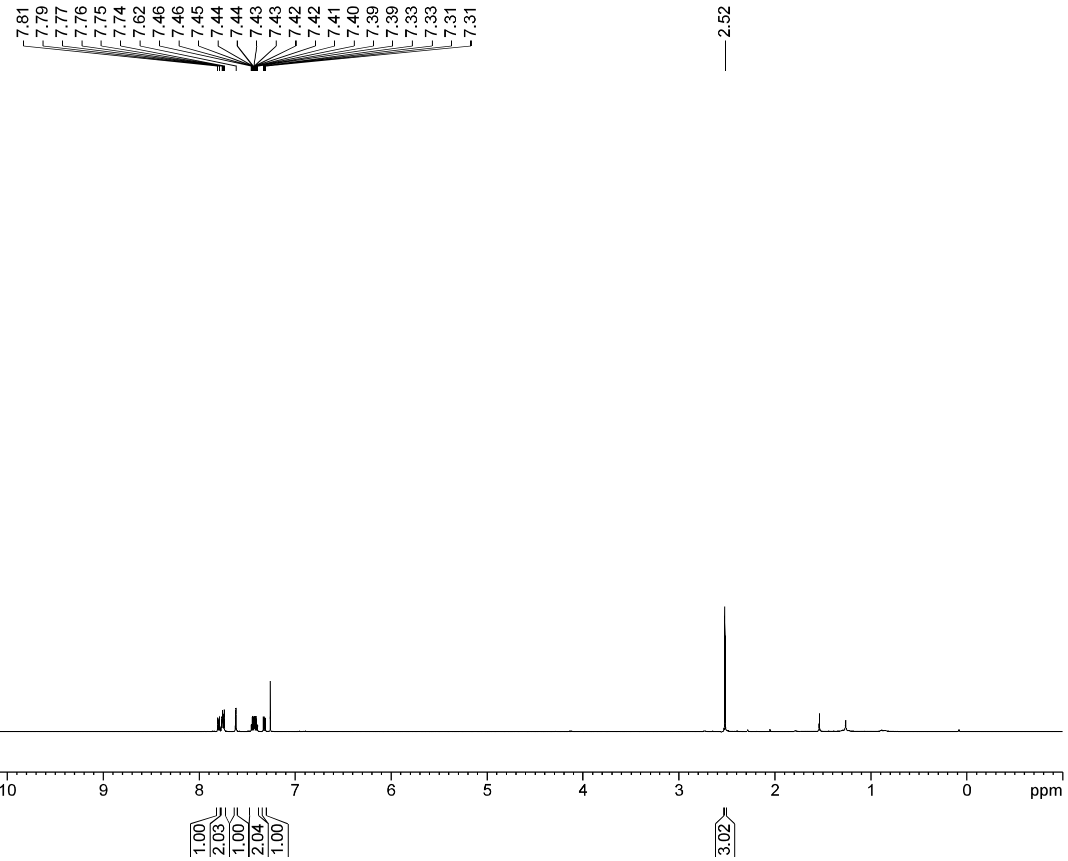

2-methyl-naphthalene

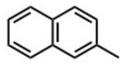

2b

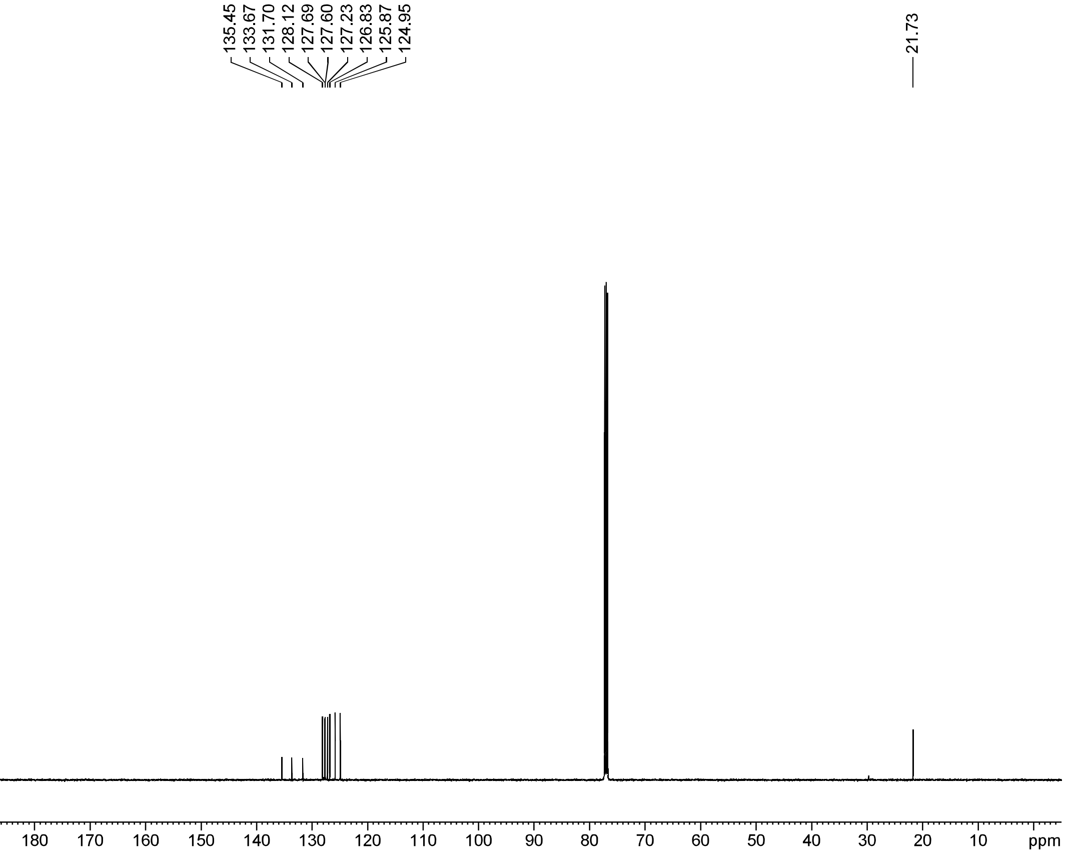

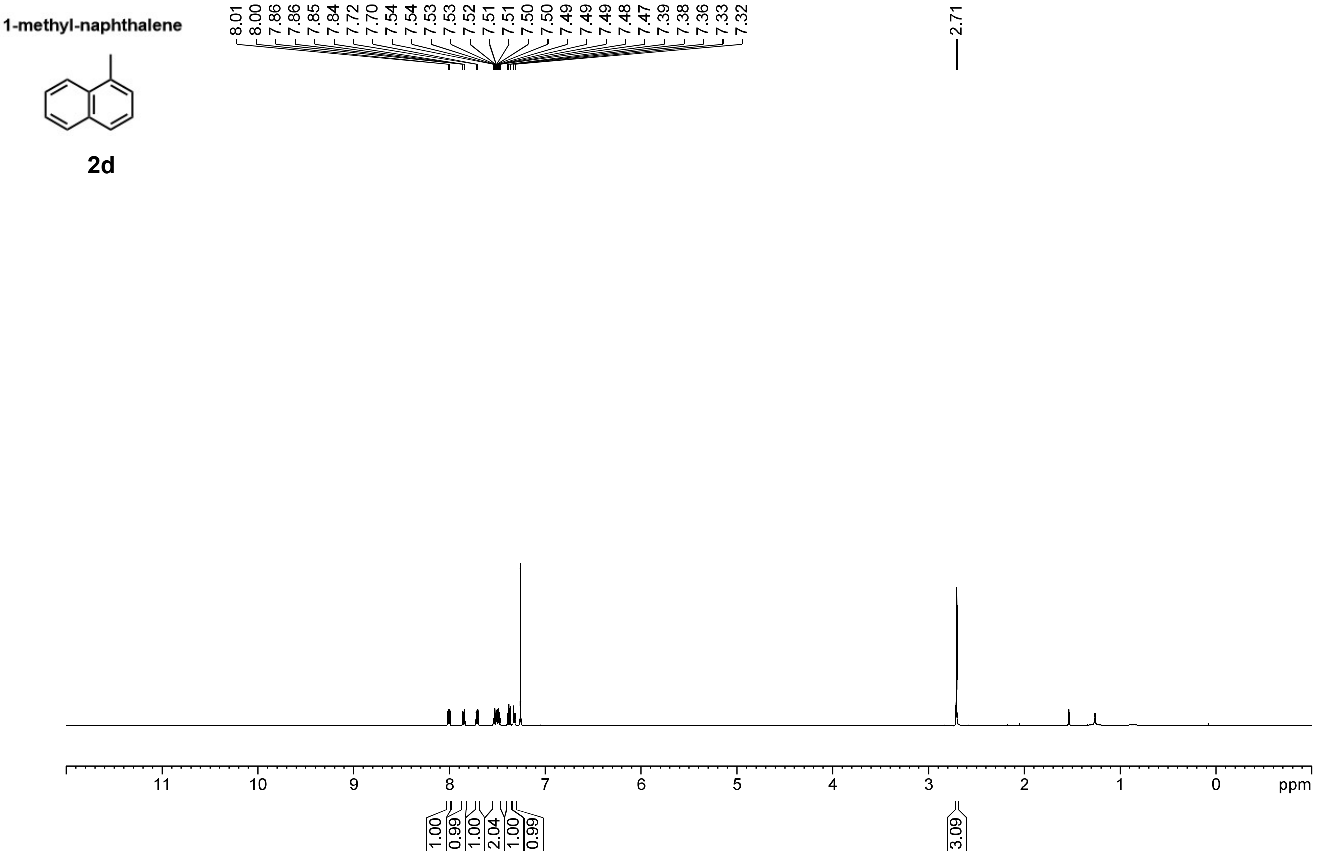
1-methyl-naphthalene
2d
8.01
8.00
7.86
7.86
7.85
7.84
7.72
7.70
7.54
7.54
7.53
7.53
7.52
7.51
7.51
7.50
7.50
7.49
7.49
7.49
7.49
7.48
7.47
7.39
7.38
7.36
7.33
7.32
2.71
11 10 9 8 7 6 5 4 3 2 1 0 ppm
1.00
0.99
1.00
2.04
1.00
0.99
3.09

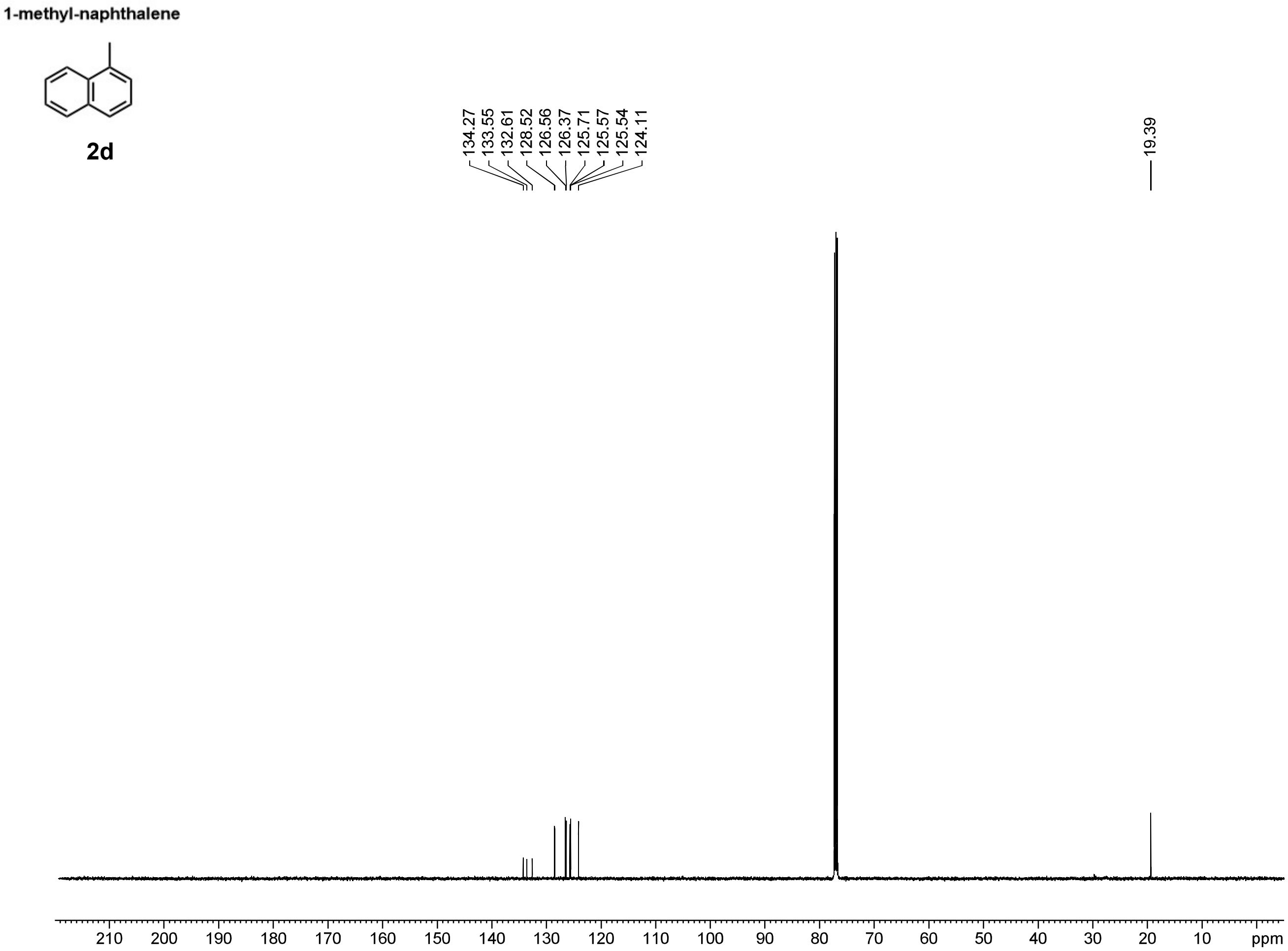
1-methyl-naphthalene
2d
134.27
133.55
132.61
128.52
126.56
126.37
125.71
125.57
125.54
124.11
19.39
210 200 190 180 170 160 150 140 130 120 110 100 90 80 70 60 50 40 30 20 10 ppm

1-methyl-naphthalene

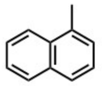

2d

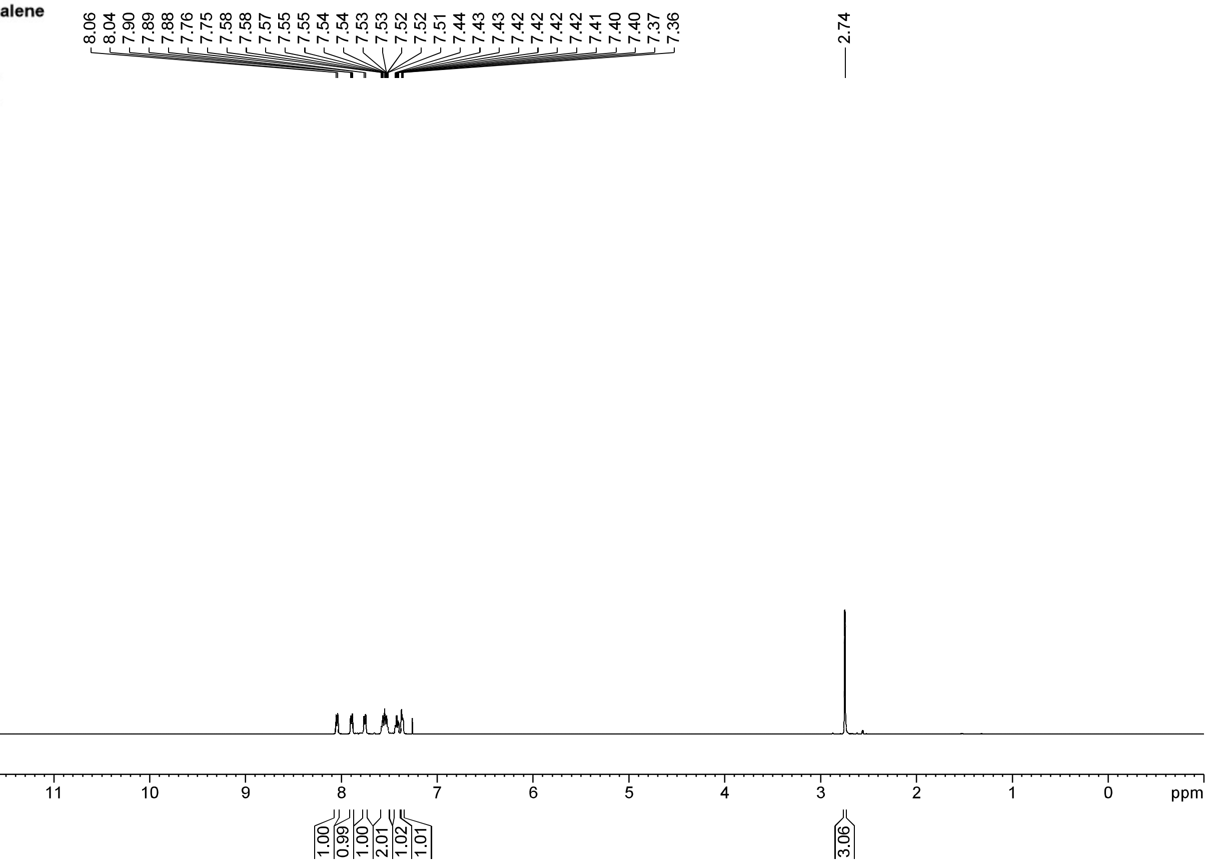

1-methyl-naphthalene

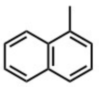

2d

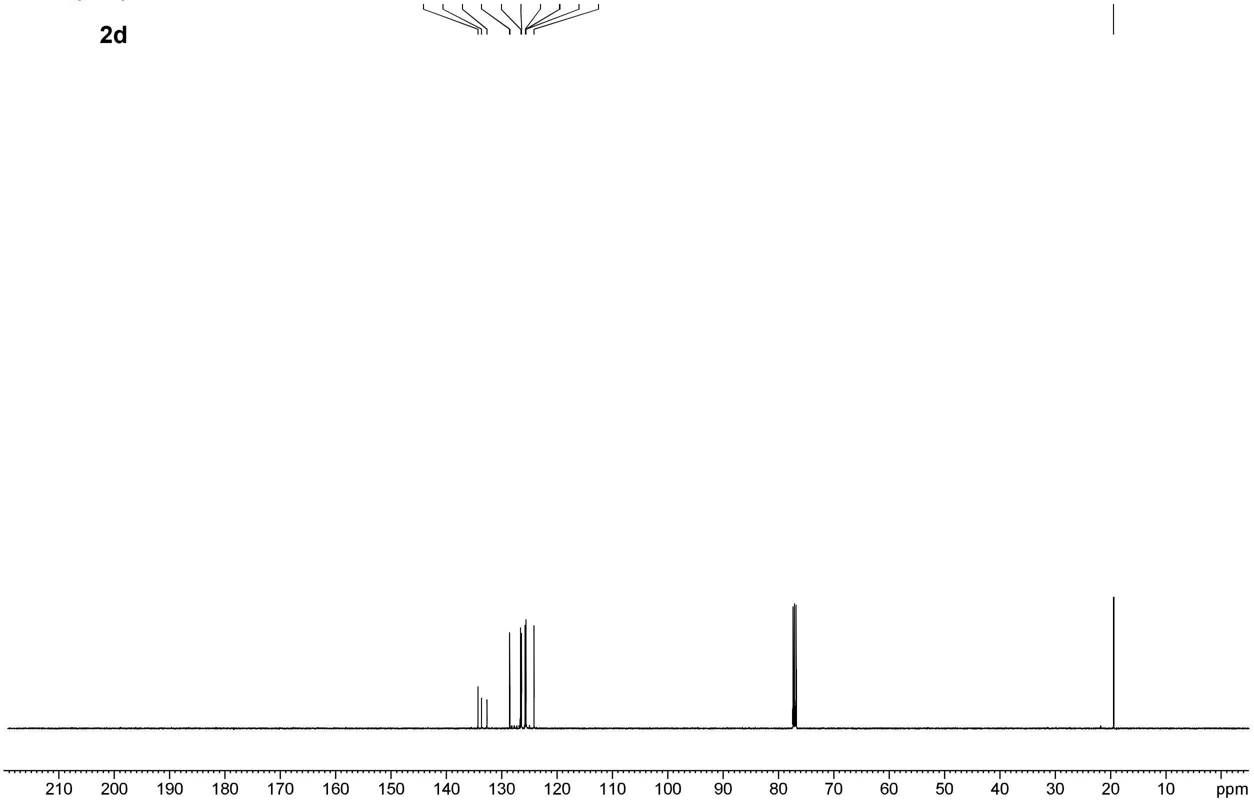

diphenylmethane

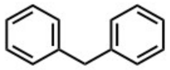

2f

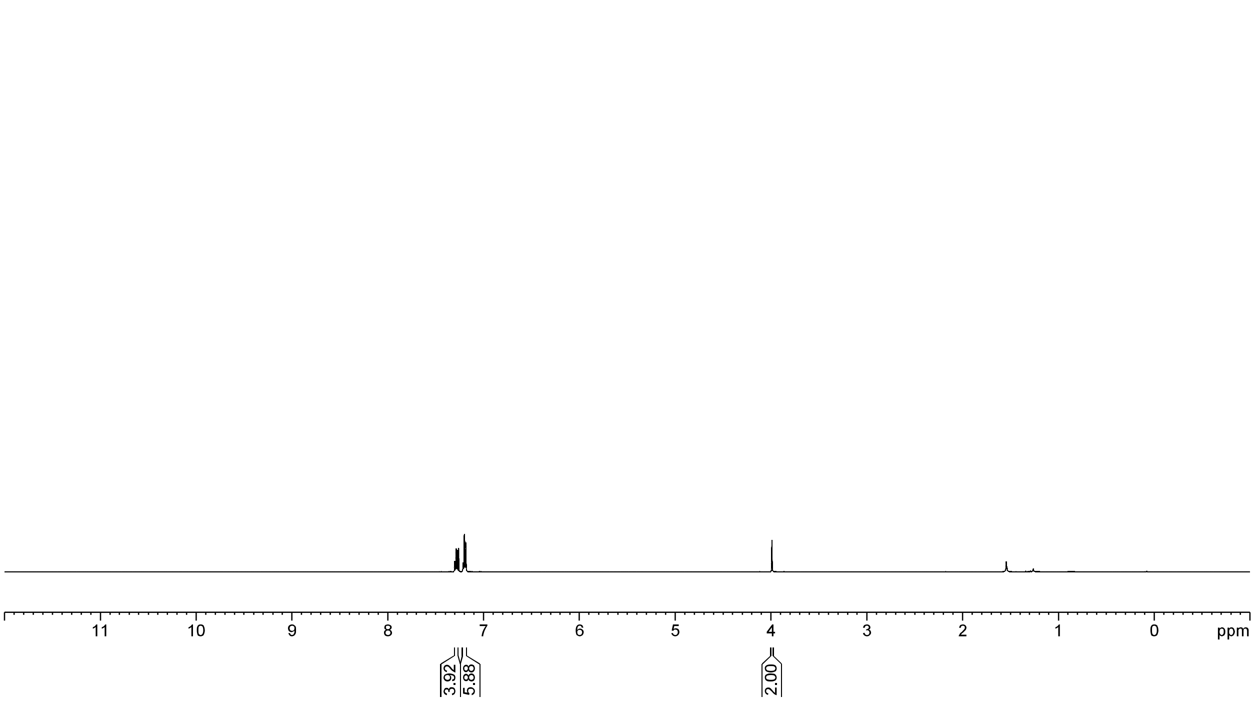

diphenylmethane

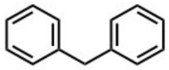

2f

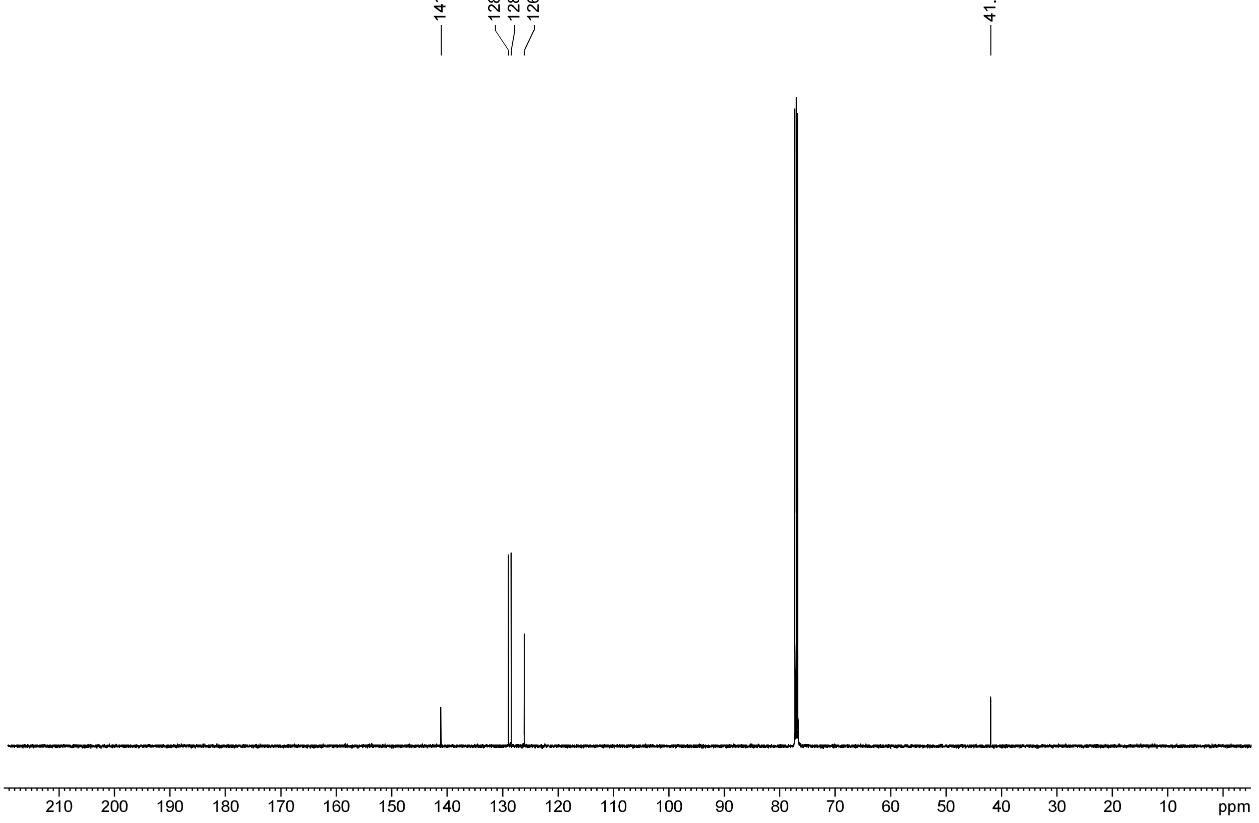

biphenyl

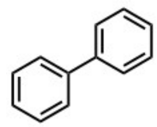

2g

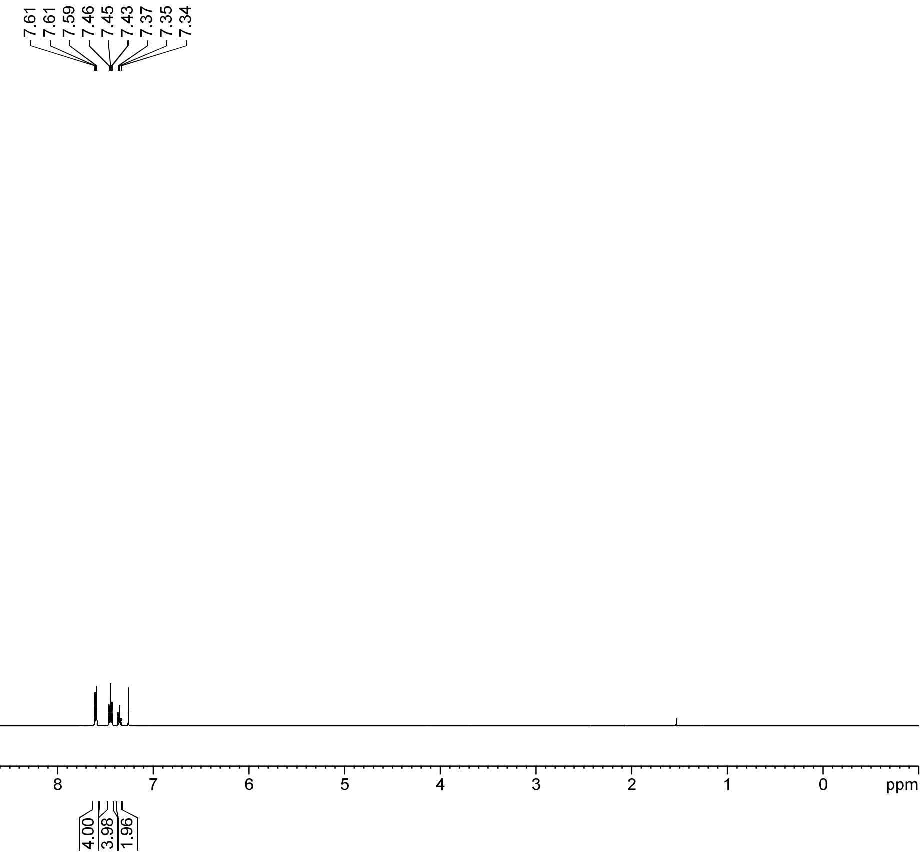

biphenyl

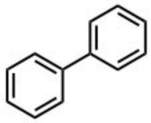

2g

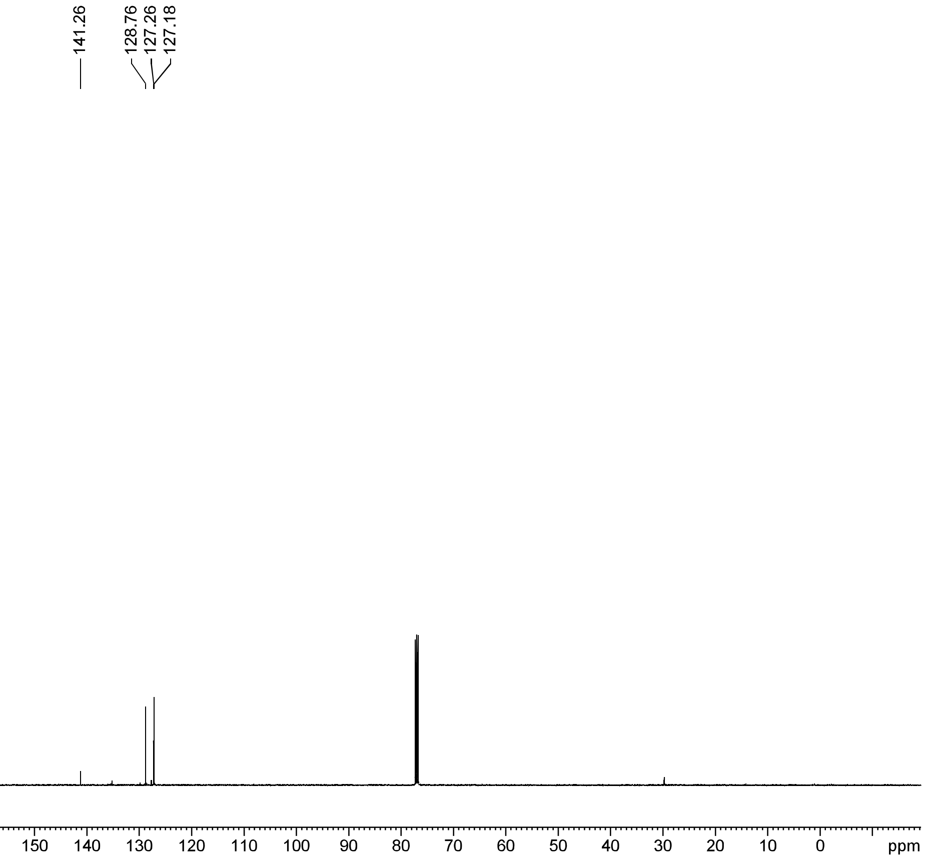

Supplement: Supplementary file 1 — Supplementary Material [file CSSC-19-e202502031-s001.pdf]
